# Genicular Artery Embolization: A New Tool for the Management of Refractory Osteoarthritis-Related Knee Pain

**DOI:** 10.3390/jpm14070686

**Published:** 2024-06-26

**Authors:** Marco Femia, Carlo Valenti Pittino, Enrico Maria Fumarola, Marco Tramarin, Maurizio Papa, Francesco Giurazza, Antonio Pio Francioso, Davide Fior, Lorenzo Paolo Moramarco, Guido Antonini, Ruggero Vercelli, Maurizio Cariati

**Affiliations:** 1 Interventional Radiology Unit, ASST Santi Paolo Carlo, 20122 Milan, Italy; 2 Complex Unit of Radiology, Department of Diagnostic, ASST Lodi, 26900 Lodi, Italy; 3 Interventional Radiology Department, AORN “A. Cardarelli”, 80100 Naples, Italy; 4 Department of Radiology, Sant’Anna Hospital, ASST Lariana, 22100 Como, Italy; 5 Department of Orthopedic and Traumatology, ASST Santi Paolo e Carlo, San Carlo Borromeo Hospital, 20122 Milan, Italy

**Keywords:** genicular artery embolization, GAE, knee osteoarthritis, knee pain

## Abstract

Osteoarthritis (OA) of the knee is a prevalent cause of chronic pain and disability, particularly affecting women. While traditionally attributed to chronic wear and tear, recent evidence highlights multifactorial pathogenesis involving low-grade inflammation and neoangiogenesis. Current therapeutic options include physical therapy, pharmacotherapy, and total knee arthroplasty (TKA). However, a subset of patients remain symptomatic despite conservative measures, necessitating the development of minimally invasive interventions. Genicular artery embolization (GAE) emerges as a promising option, targeting neovascularization and inflammatory processes in OA. This paper reviews the pathophysiological basis, patient selection criteria, procedural details, and outcomes of GAE. Notably, GAE demonstrates efficacy in relieving knee pain and improving function in patients refractory to conventional therapy. While further research is warranted to elucidate its long-term outcomes and compare it with existing modalities, GAE represents a novel approach in the management of symptomatic knee OA, potentially delaying or obviating the need for surgical intervention. Here, we synthesize the relevant literature, technical details of the procedure, and future perspectives. Moreover, the success of GAE prompts the exploration of transarterial embolization in other musculoskeletal conditions, underscoring the evolving role of interventional radiology in personalized pain management strategies.

## 1. Introduction

Osteoarthritis is a leading cause of adult chronic pain and disability, with the knee representing the most common site of osteoarthritis [1]. It has an age standardized prevalence of about 4300 cases per 100,000 individuals, with women being more affected than men [2].

Although chronic “wear and tear” is traditionally thought to be the main mechanism, new evidence suggests that the pathogenesis of knee osteoarthritis is multifactorial, with low-grade inflammation and neoangiogenesis playing a key role [3]. The treatment armamentarium for the management of symptomatic knee osteoarthritis (KO) includes physical therapy, drugs, such as non-steroidal anti-inflammatory drugs (NSAIDs), intra-articular infiltrations of hyaluronic acid and corticosteroids, and, eventually, total knee arthroplasty (TKA). A broad group of patients remain symptomatic despite therapeutic efforts, with a subset showing mild to moderate articular degeneration which would not warrant surgical joint replacement [4]. Thus, there is a need to develop new, effective, minimally invasive treatments to address symptomatic mild to moderate KO resistant to conservative therapies.

## 2. Pathophysiology

### 2.1. Inflammation

Inflammation can be either acute or chronic, when long-lasting, in response to injury.

While acute inflammation is locally characterized by fluid effusion with granulocytic cells, accompanied by systemic responses such as fever, leukocytosis, protein catabolism, and serum C-reactive protein elevation, chronic inflammation is usually more subtle and characterized by tissue monocytic infiltration [5,6,7].

Acute inflammation may resolve or evolve into chronic inflammation while the latter can also start directly from the very beginning [6,8].

### 2.2. Angiogenesis

The synovial joint has a structure which allows both stability and movement. The normal synovial lining is highly vascular, feeding the avascular articular cartilage [9].

Angiogenesis is the development of new capillaries from pre-existing blood vessels. Inflammatory cells produce proangiogenic factors which stimulate the growth and invasion of new blood vessels, which ease inflammatory cell infiltration. In osteoarthritis, angiogenesis seems to maintain rather than trigger inflammation [7].

Angiogenesis results from a cascade of processes and is involved in different pathologic processes such as chronic inflammation and the metastatic spread of tumors.

Angiogenic factors, such as vascular endothelial growth factor (VEGF), angiopoietin-1 (Ang-1), nerve growth factor (NGF), and basic fibroblast growth factor (bFGF), released within the synovium by many inflammatory cells, stimulate local endothelial cells in secreting proteolytic enzymes, which degrade the endothelial basement membrane and the perivascular extracellular matrix. This allows endothelial cells to proliferate and migrate to develop a primary sprout which, in turn, leads to the development of capillary loops. The subsequent synthesis of a basement membrane around the vascular loops ultimately leads to capillary formation [10].

The microenvironment of inflammatory areas of the synovial membrane seems to have higher levels of pro-inflammatory cytokines than normal areas. In fact, researchers demonstrated a higher secretion of pro-inflammatory mediators, such as VEGF, interleukin-6 (IL-6), interleukin-8 (IL-8), by synovial fibroblast cells of inflammatory areas and a lower secretion of trombospondin-1, which is an anti-angiogenic factor [11].

Pro-inflammatory cytokines such as IL-6, IL-8, and tumor necrosis factor-a (TNF-a) are involved in OA as well as in neovascularization, either directly stimulating the process or promoting the secretion of VEGF [12]. In regard to neovascularization, VEGF seems to play a key role in the angiogenic process, promoting both the proliferation and migration of endothelial cells [13].

The final effect of these mediators is an increased vascularity in the synovium seen in OA patients and an invasion of cartilage by new vessels from the underlying bone [14]. As a result, patients with OA of all grades experience a thickening of the synovial lining cell layer, neovascularization, and inflammatory cell infiltration of the synovial membranes, changes that are more evident in severe OA [14,15]. The awareness of the importance of the angiogenetic process stimulated the need to develop new lines of research which addressed the treatment of neovessels in the osteoarthritic joints, leading to the development of the genicular artery embolization (GAE) procedure.

## 3. Indications and Patient Preparation

The therapeutic efforts of painful knee osteoarthritis traditionally focused on physical therapy (such as muscle strengthening, exercises, and stretching), anti-inflammatory drugs (such as NSAIDs, corticosteroids, and opioids) and intra-articular infiltrations of hyaluronic acid and corticosteroids. However, the last traditional option for patients not responding to these therapies is TKA [4]. The non-invasive nature of physical therapy justifies its role as the first line therapy for the disease. When ineffective, patients start medications and interventions. NSAIDs often provide minimal pain relief and their chronic overuse raises the risk of kidney and hepatic failure; moreover, opioids may induce addiction and corticosteroids give a high risk of both metabolic impairment and osteoporosis. Intra-articular injections of hyaluronic acid and corticosteroids, although minimally invasive, often provide limited efficacy over the long term, thus requiring repetitive sessions [4,16]. Due to the longer life expectancy of patients, the objective of the genicular artery embolization procedure is to postpone surgical replacement as much as possible, or to offer another therapeutic chance to those patients not willing to undergo or not eligible for TKA.

The main case series include young or older adults with moderate to severe knee pain (i.e., a visual analogue scale (VAS, from 0 to 10) higher than 4) who did not experience benefit from at least 3 months of continuous conservative therapies [17,18,19,20]. Moreover, the assessment of the Western Ontario and McMaster Universities Osteoarthritis Index (WOMAC) preoperatively is suggested to understand the baseline symptoms and compare the result with those obtained after the treatment to understand its effectiveness [21,22].

**Figure 1:**
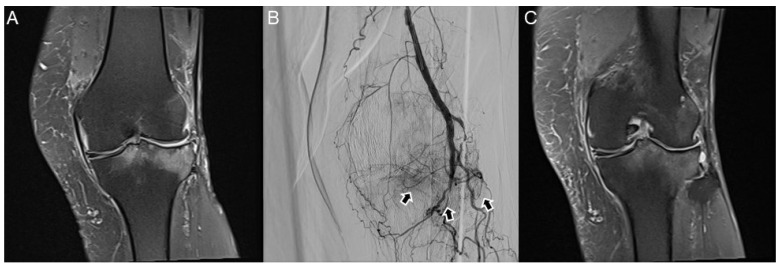
Magnetic resonance imaging and angiographic findings in an 87-year-old patient affected by osteoarthritis. The patient had undergone multiple conventional therapies over the years, including joint infiltrations and oral analgesics. She was not considered a candidate for TKA due to high surgical risk. Coronal fat-saturated T2-weighted MRI (**A**) shows diffuse oedema in the lateral compartment of the tibia. DSA performed at the femoral-popliteal transition (**B**) shows an area of hypervascularisation (blush) in the lateral compartment of the tibia (arrows) corresponding to the MRI finding. Magnetic resonance imaging conducted 4 months post embolization (**C**) exhibits a notable attenuation of bone marrow oedema; clinically, the patient reported diminished symptomatic pain and achieved unaided ambulation.

To be proposed for GAE, patients need to undergo plain radiography in order to assess their Kellgren–Lawrence (KL) grade. KL grading is a five-grade system, published in 1957, based on plain radiography, which evaluates the severity of articular osteoarthrosis changes [23]. Usually, eligible patients include KL grade 1 to 3, while KL grade 4 patients are proposed for TKA unless they refuse or they are considered inoperable; in such cases, GAE may be proposed to improve symptoms although the literature on high-grade osteoarthritis is still insufficient. Pre-operative magnetic resonance imaging (MRI) without contrast medium (Figure 1) is suggested to exclude other sources of knee pain [20,24]; however, some authors suggest to complete the MRI study with post-contrast T1-weighted sequences [19].

The additional value of post-contrast MRI images is to identify the areas of synovial hypervascularization, with the possibility to match them with clinical data and intraprocedural findings in order to guide the embolization procedure [25].

Peripheral arterial disease (PAD) represents one of the main contraindications because geniculate arteries play an important role as collateral circulation in case of popliteal and below-the-knee artery atherosclerotic occlusion, and patients with PAD may rely on these vessels to sustain leg perfusion [26]. In addition, severe PAD may complicate the procedure due to eventual genicular artery stenosis with a higher risk of arterial dissection, especially when treating those arteries branching off perpendicularly. Thus, a careful evaluation of peripheral pulses is mandatory while arterial duplex ultrasound is usually reserved to doubtful situations. It is also important to rule out general contraindications to arteriography by investigating baseline renal and coagulative function and possible allergy to iodinated contrast medium. Finally, due to the relatively high percentage of transient skin mottling occurrence, it is important to perform a pre-intervention dermatologic evaluation of the knee region.

## 4. Local Vascular Anatomy

The genicular arteries are a network of vessels that supply blood to the knee joint region.

**Figure 2:**
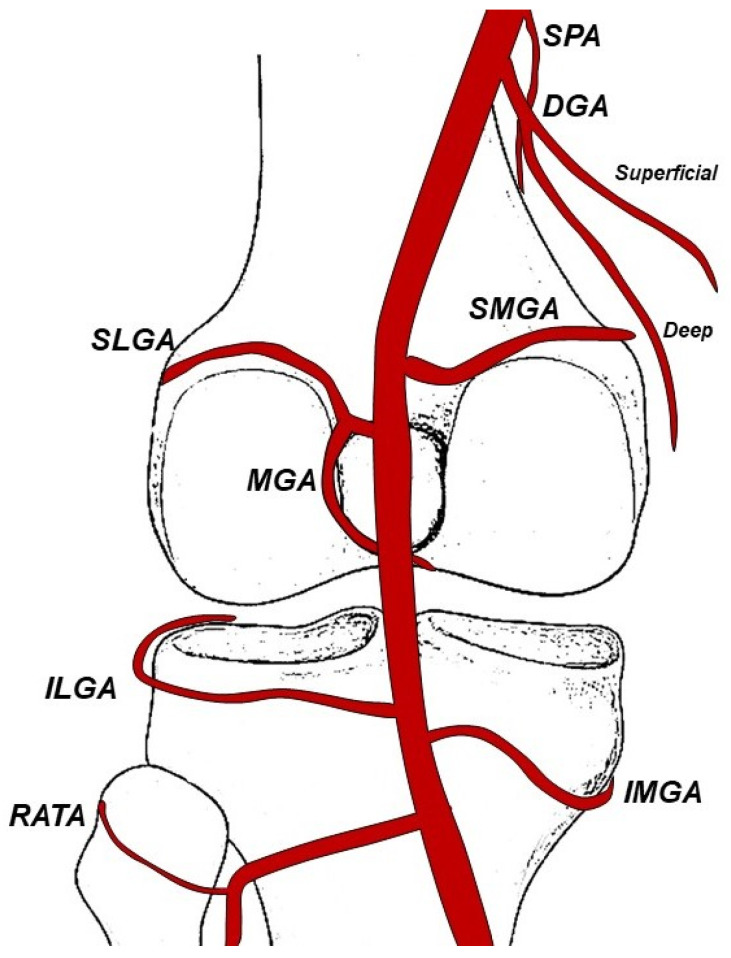
The typical branching patterns of the genicular arteries.

These arteries include the descending genicular artery (DGA), superior lateral genicular artery (SLGA), superior medial genicular artery (SMGA), middle genicular artery (MGA), inferior lateral genicular artery (ILGA), inferior medial genicular artery (IMGA), recurrent anterior tibial artery (RATA), and superior patellar artery (SPA). The conventional genicular artery anatomy is illustrated in the figure below (Figure 2).

The medial portion of the knee receives its primary blood supply from the DGA, SMGA, and IMGA. The DGA originates from the superficial distal femoral artery, and bifurcates into a superficial (myocutaneous) branch, known as the superficial medial saphenous branch, and a deeper, more convoluted lateral musculoarticular branch, that perfuses the joint.

The SMGA divides into one branch supplying the vastus medialis, while the other branch supplies the femur and knee joint.

The IMGA supplies the upper end of the tibia and knee joint.

The MGA may originate from a common point with the LSGA or isolated. It provides perfusion to both the anterior and posterior cruciate ligaments and to the synovial membrane of the knee.

The SPA has a variable origin from the DGA or directly from the femoral artery and has a characteristic serpiginous course to the anterior part of the knee, toward the patellofemoral compartment.

The lateral compartment of the knee is mainly perfused by the SLGA, ILGA, and RATA. The SLGA is divided into a superficial branch that supplies the vastus lateralis and a deep branch that supplies the lower part of the femur and knee joint.

The ILGA ends with branches that anastomose with the IMGA, SLGA, and RATA [22].

The medial joint compartment is the weight-bearing portion of the joint and therefore the one most frequently affected by degenerative osteoarthritis [27]. Consequently, the arterial branches of this compartment are most often targeted for embolization.

Significant variation in genicular artery anatomy exists and holds crucial significance for interventional radiologists. A recent cadaveric study [28] provided a comprehensive classification system for variations in branching patterns and the presence of arterial anastomoses. This thorough understanding of anatomy is paramount for ensuring the safety and efficacy of interventions.

The presence of arterial anastomoses is particularly noteworthy during embolization procedures due to the potential risk of non-target embolization via these connections. In their study, O’Grady et al. [28] observed that the SMGA shared an origin with the MGA in 25% of cases. Non-target embolization (NTE) to the MGA may result in damage to the cruciate ligaments. In addition, anastomosis between the DGA and SMGA was found in 85% of cases, with a mean diameter of 850 microns, representing another risk of NTE.

Intraoperative cone-beam computer tomography (CBCT) stands as a crucial supplement to conventional angiography and CT angiography, providing a more comprehensive understanding of genicular artery anatomy. In comparison to digital subtraction angiography (DSA), CBCT has superior resolution capabilities, which allow it to accurately identify complex structures such as small-caliber vessels and anastomoses [29]. The high anatomic definition offered by CBCT plays a key role in interventional planning and in ensuring the accuracy and safety of procedures targeting the genicular arteries.

In their study, E. Callese et al. [29] identified through CBCT four genicular artery branching variants: Type 1: common trunk of SLGA and MGA; Type 2: independent origins; Type 3: common trunk of the SLGA, MGA, and SMGA; and Type 4: common trunk of the MGA and SMGA.

Understanding the anatomy and variants of the geniculate arteries is essential for interventional radiologists performing procedures such as GAE, in terms of treatment efficacy and to minimize complications.

## 5. Embolization Procedure

GAE is an elective symptom-related procedure which implies the treatment of painful regions showing “tumor blush” appearance at selective and/or superselective angiography. As such, it is important to ask the patient to point to the most painful sites in order to place radiopaque markers on the skin, useful to guide the embolization.

Before the procedure, intravenous antibiotic prophylaxis should be administered (commonly, 2 g of cephazolin). The angiographic suite should be prepared for peripheral angiography, so it could be useful to reverse head-to-feet the usual patient position.

Ultrasound-guided vascular access is recommended and it may be performed either antegrade or contralateral retrograde, in case of a prominent belly or atherosclerotic plaques on the common femoral artery.

The introducer sheath can be selected on the basis of the operator preference. The most common introducer sheaths used are standard 4 French sheath and the 3 French sheath, commonly included in the micropuncture set. In the latter case, the operator renounces the use of an intermediate 4 French diagnostic catheter.

Then, a digital subtraction angiography (DSA) of the superficial femoral artery at the knee level is performed by injecting either through the sheath or through the diagnostic catheter 15 mL of iodinated contrast agent at a flow rate of 3 to 4 mL/s. It is important to make a long-lasting angiographic run to observe the parenchymal phase. “Tumor blush” may not be visible through the femoral or popliteal injection, so a superselective angiography of the arteries supplying the painful region is necessary. Superselective catheterization should be performed with a 2.4 Fr or thinner microcatheter in order to facilitate a distal embolization avoiding spasm.

It is important to identify dangerous anastomoses such as those with popliteal artery, or other collaterals which can lead to off-target embolization, such as cutaneous collaterals. In this setting, the use of cone-beam computed tomography (CBCT) is helpful due to its three-dimensional view and its higher resolution compared to the two-dimensional images of DSA.

**Figure 3:**
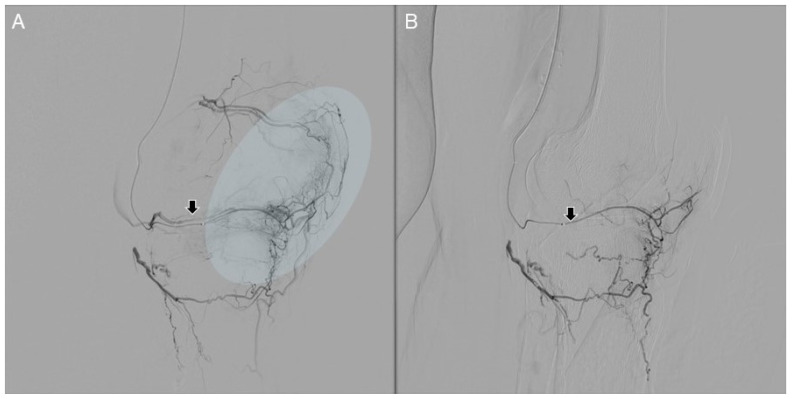
Selective angiography of the ILGA using a microcatheter (arrow). Initial angiographic image (**A**) shows hypervascular “blush” (highlighted in blue oval). After the delivery of 100–300 micron microspheres, the angiogram (**B**) shows “pruning” of the artery with patent parent vessel. The patient had no procedural adverse events.

Before starting the embolization, an ice pack should be placed on the skin of the target region. This significantly reduces the risk of skin ulceration by inducing the vasoconstriction of skin collaterals [20]. Once the microcatheter is advanced within the target vessel, a DSA is necessary to demonstrate the “tumor-like blush” appearance, and the embolic material is slowly injected until pruning of the neovascularity is obtained and pathologic hyperemia is no longer seen, while preserving the patency of the parent vessel (Figure 3).

As the amount of embolic material is usually small, it is suggested to inject 0.2 increments of embolic material, followed by saline. In addition, between each bolus, DSA is necessary to recognize the appropriate embolization point. Every vessel supplying the painful region should be studied and treated if “tumor-like blush” is recognized. Finally, the hemostasis of the vascular access can be achieved either through an occlusion device or manual compression.

### Embolic Materials

The first case series about the treatment of refractory knee pain secondary to osteoarthritis, published by Okuno et al., described the use of both temporary and permanent embolic agents.

Okuno and colleagues employed imipenem/cilastatin sodium as a temporary embolic and permanent 75 mm microparticles.

Imipenem/cilastatin is an antibiotic which demonstrates slight solubility in water, and, when suspended in contrast agent, constitutes 10 to 70 mm particles that have an embolic effect [17].

The results of Okuno et al. showed the good efficacy of both embolic materials without major adverse events [17].

Unfortunately, imipenem/cilastatin sodium is not readily available everywhere with this indication. As such, many authors focused on the use of microparticles with good results but obtaining a permanent embolization [17,18,19,20].

However, the possibility to use a temporary embolic agent seemed a great opportunity in order to reduce the risk of ischemic adverse events. In a recent trial by Sapoval and colleagues, the authors decided to study the efficacy of an ethiodized oil-based emulsion between the temporary embolic agent Lipiodol Ultra-Fluid (Guerbet) and the contrast agent ioversol 300 mgI/mL in a 1:3 mixture which was found in a previous reserved study, by their group, to be able to embolize vessels for about 10 min. In their LipioJoint-1 trial, they obtained good clinical results and no embolic material-specific adverse events. At the same time, Min et al. tested another temporary embolic material based on the gelatin sponge widely used for the temporary embolization of bleeding vessels or at the end of hepatic chemoembolization to obtain stasis within the treated vessels, which is said to be degraded within 24–48 h into the bloodstream [30]. A recent metanalysis compared the performance of temporary and permanent embolic agents. The results did not show significant differences in patients’ outcomes between the two groups [31]. However, to date, the experience is still limited and there is no wide consensus yet on which embolic material is safer and more efficacious, although results on both matters seem to be comparable.

## 6. Expected Outcome and Complications

### 6.1. Expected Outcome

The results of GAE for the treatment of refractory knee pain secondary to osteoarthritis are usually evaluated with the use of validated self-administered scales either general, such as VAS, or specific for hip and knee osteoarthritis as in the case of WOMAC. While the former exclusively evaluates pain, WOMAC also takes into consideration articular functionality and stiffness. Some authors decided to use the Knee Injury and Osteoarthritis Outcome Score (KOOS), which is a more recent questionnaire evaluating symptoms related to osteoarthritis and their impact on patients’ quality of life [32,33,34].

**Figure 4:**
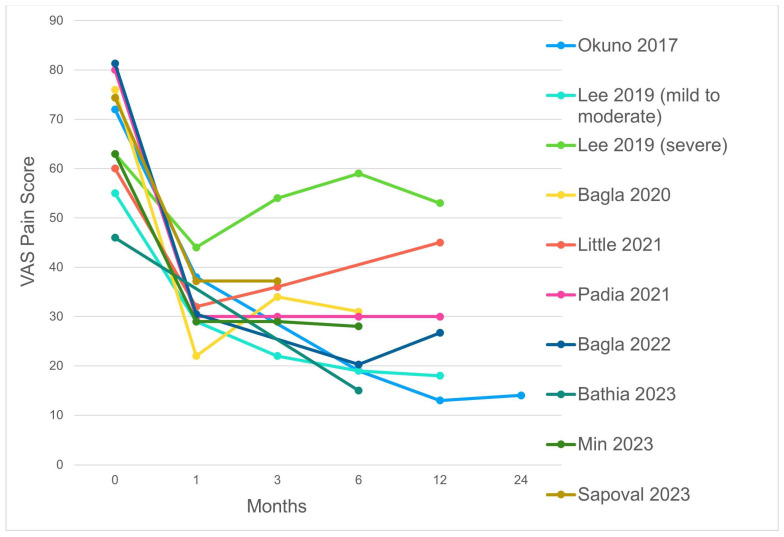
VAS score during the follow-up period of the main case series [18,19,20,30,35,36,37,38].

**Figure 5:**
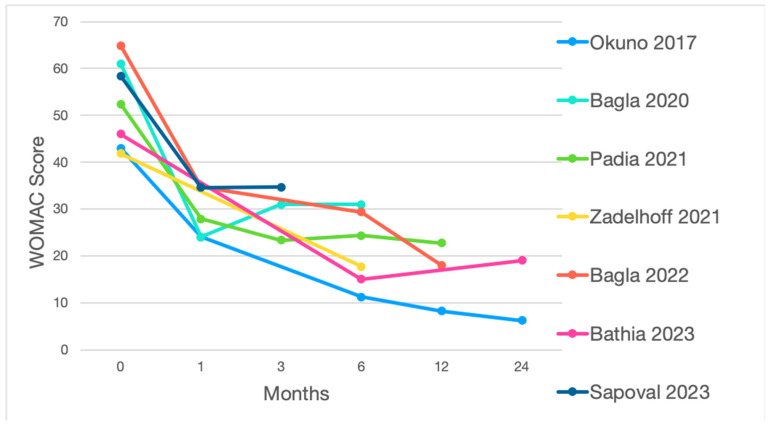
WOMAC score during the follow-up period of the main case series [19,20,36,37,38,39,40].

In the main case series published so far, both VAS and WOMAC scores were substantially reduced within the first month [17,18,19,20,35,36,37], often remaining low until the third month (Figure 4 and Figure 5).

However, in some papers, a small rebound in pain and articular symptoms was registered after the first month [18,19,35]. This finding can be due to the patient’s characteristics. In the paper by Lee et al. only the subset affected by severe osteoarthritis experienced symptom rebound, while the subset of patients with mild to moderate KO had a long lasting benefit which remained up to 12 months, suggesting a poorer outcome in more advanced KO [35]. Choi and colleagues observed a significant difference in response among KL grades, identifying a grading ≥3 as a predictor of poor response [24], and thus highlighting the importance of OA severity. Nevertheless, these results are in contrast with the subsequent experience by Padia and colleagues, whose study population included up to 40% of KL grade 4 patients. In their study, 60% and 53% of patients registered a decrease of at least 50% in VAS and WOMAC scores, respectively, at 1 month, and the percentage rose up to 68% in both categories at the 1-year follow-up [20]. The main difference between these experiences is that while the group of Padia obtained vessel embolization through non-resorbable particles, Lee et al. and Choi and colleagues employed IM/CS and a combination of IM/CS and gelatin sponge, which are all temporary embolic agent. Finally, the most recent case series published by Sapoval and colleagues employed another type of resorbable embolic agent based on the ethiodized oil Lipiodol Ultra-Fluid (Guerbet, Paris, France) [39]. They exclusively enrolled KL 3 and 4 patients, obtaining a mean reduction in VAS of 50% for the three-month follow-up. Although promising, this result needs to be confirmed over the long term.

In conclusion, based on the most recent studies, patients affected by OA-related knee pain can expect a 30% to 50% reduction in VAS and WOMAC scores, which in most cases lasts up to 6 months, and can protract up to 1 or 2 years. The effectiveness of GAE is less predictable in more severe settings, particularly in KL 4 grades, for which research results are still poor and controversial.

### 6.2. Complications

Most of the adverse events in GAE are secondary to non-target embolization. Indeed, those most observed across the main case series published so far are skin mottling and discoloration, which can occur in up to 65% of cases [19,30], and this is thought to be due to the embolization of small perforators supplying the skin, which cannot be avoided. Due to the undesired embolization of cutaneous branches, Padia et al. [20] reported seven cases of focal skin ulceration (<1 cm), which resolved spontaneously without sequelae. Since the group decided to adjust the embolization protocol by applicating an ice pack over the target area before the procedure, no more events of skin necrosis occurred. At the 3-month MRI, the same group observed 2 cases out of 40 enrolled patients of clinically asymptomatic focal bone infarct smaller than 2 cm.

Two studies reported three cases of numbness in the below-the-knee area, which resolved in two weeks after the administration of gabapentin [19,36]. Although the embolization is usually minimal, Lee et al. registered one case of mild fever which spontaneously subsided in 1 day [35].

Finally, arterial access can be a source of complications as in other endovascular interventions. Although some cases of puncture site hematomas are exclusively reported in the literature [17,19,20,30,34,35], it is always necessary to pay attention to antegrade arterial punctures, which can give origin to arterial dissection and occlusion.

## 7. Transarterial Embolization in Other Musculoskeletal Conditions

The application of GAE also demonstrated successful results in the setting of recurrent hemarthrosis after TKA. This is an uncommon condition (0.3–1.6%); however, it is usually refractory to conservative treatment, and surgical revision is often required to obtain a definitive resolution [41,42]. GAE offers a minimally invasive solution with high rates of clinical success ranging from 76% to 89%, occasionally requiring more than one procedure [43].

Beyond knee-related conditions, a wide variety of musculoskeletal disorders can lead to chronic musculoskeletal pain, such as low back pain, hip pain, and rheumatoid and osteoarthritis (OA)-associated joint pain. The main symptom in patients with chronic musculoskeletal disorders is intense pain, which is often accompanied by stiffness. This frequently leads to sleep alterations, fatigue, reduced activity, mood impairment, and severe disability, with a significant impact on the quality of life.

Transarterial embolization (TAE) has been employed over time to treat many other pathologic musculoskeletal conditions, although data on its application in different joints and tendons are still very limited [44]. Firstly, TAE for musculoskeletal pain was employed by Okuno and colleagues to treat tendinopathy and enthesopathy refractory to nonsurgical management [45], even earlier than its application for the treatment of knee OA. They studied TAE in seven patients with tendinopathy and enthesopathy located at different sites (one patellar tendinopathy, two rotator cuff tendinopathies, one plantar fasciitis, one lateral epicondylitis, one iliotibial band syndrome, and one Achilles insertion tendinopathy), all united by recalcitrant pain. They registered a reduction in VAS scores greater than 85% for the entire follow-up period (4 months). The impressive success of this experience encouraged other groups to evaluate this technique in other musculoskeletal painful conditions. One of the most successful sites was the hip joint, with a great clinical success reported in a case report by Correa and colleagues [46], who registered complete pain resolution the day after the procedure, which was maintained up to the 4-month follow-up visit. Two years later, the same group published a more extensive experience on the treatment of painful hip joint, confirming the efficacy of TAE in the control of hip pain related to osteoarthritis and greater trochanteric pain syndrome [47]. Finally, TAE was also successfully employed in trapeziometacarpal and finger osteoarthritis, shoulder osteoarthritis, adhesive capsulitis, and many other conditions.

## 8. Future Directions and Conclusions

Genicular artery embolization (GAE) stands at the forefront of a new era in knee pain management, offering a personalized therapeutic approach through collaboration between interventional radiologists and orthopedic surgeons. In the treatment of refractory knee pain secondary to osteoarthritis (OA), GAE presents a promising alternative for patients who have exhausted conventional minimally invasive options, avoiding the necessity for suboptimal surgical interventions or enduring chronic pain.

The efficacy of GAE in alleviating knee pain is underscored by its impact on validated self-administered scales, such as the Visual Analog Scale (VAS) and the Western Ontario and McMaster Universities Osteoarthritis Index (WOMAC). Studies have shown significant reductions in pain and improvement in joint functionality, often sustained for months following the procedure. However, variability in outcomes exists, particularly in more severe cases of OA, emphasizing the need for further research to identify optimal candidates and standardize technical protocols.

Future investigations should focus on elucidating the long-term durability of pain relief, functional improvement, and potential adverse events associated with GAE. Additionally, understanding the procedure’s impact on joint health and its role in the progression of osteoarthritis is paramount. Establishing comprehensive pre-operative evaluation criteria and follow-up protocols will not only optimize patient outcomes but also minimize unnecessary costs.

A holistic comparison between GAE, traditional pharmacotherapy, intra-articular injections, and surgery could provide invaluable insights into the most appropriate treatment modality for knee osteoarthritis. Furthermore, exploring GAE’s potential to interrupt the vicious cycle of neoangiogenesis and inflammation may hold the key to slowing disease progression.

Finally, the success of GAE should encourage researchers to study the role of TAE in the management of other painful musculoskeletal conditions in order to legitimize its application as a frontline therapy of chronic musculoskeletal pain.

In conclusion, GAE heralds a paradigm shift in knee pain management, offering patients a ray of hope for improved quality of life through less invasive and more targeted interventions. As research advances and clinical experience grows, GAE has the potential to revolutionize the therapeutic landscape, providing a tailored approach to each patient’s unique needs and circumstances.

## Data Availability

Not applicable.
